# Entrapment of Hydrophobic Biocides into Cellulose Acetate Nanoparticles by Nanoprecipitation

**DOI:** 10.3390/nano10122447

**Published:** 2020-12-07

**Authors:** Cynthia Cordt, Tobias Meckel, Andreas Geissler, Markus Biesalski

**Affiliations:** Macromolecular and Paper Chemistry, Technical University of Darmstadt, Alarich-Weiss-Straße 8, 64287 Darmstadt, Germany; cordt@cellulose.tu-darmstadt.de (C.C.); meckel@cellulose.tu-darmstadt.de (T.M.)

**Keywords:** nanoparticles, nanoprecipitation, entrapment, cellulose acetate, cellulose ester, solvent replacement, biocide, drug loading, antimicrobial paper, packaging

## Abstract

This contribution reports an efficient method for the production and use of biocide-loaded cellulose acetate nanoparticles. As well-known model biocides 4-Hexylresorcinol and Triclosan were used for in situ nanoparticle loading during a nanoprecipitation process. We show that the nanoparticle size can be well-controlled by variation of the cellulose acetate concentration during nanoprecipitation. Apart from strong evidence suggesting cellulose acetate particle formation according to a nucleation-aggregation mechanism, we further show that the biocide loading of the particles occurs by a diffusion process and not via co-precipitation. The quantity of particle loading was analyzed by ^1^H-NMR spectroscopy of re-dissolved nanoparticles, and it was observed that a decisive factor for high packaging efficiency is the use of a biocide with low water solubility and high hydrophobicity. SEM studies showed no influence on the particle morphology or size by both biocides 4-Hexylresorcinol and Triclosan. Finally, an aqueous nanoparticle dispersion can be coated onto model paper sheets to yield pronounced antimicrobial surface-properties. Nanoparticles loaded with the biocide Triclosan showed a high antimicrobial activity against *Bacillus subtilis*, a cellulase producing bacteria, if applied to model paper substrates, even at extremely low coating weights of 1–5 g/m^2^, respectively. Additional long-term efficacy renders these nanoparticles ideal for various applications.

## 1. Introduction

Polymeric nanoparticles loaded with small functional molecules are widely known in medical applications but also in other fields such as cosmetic, agricultural or food industry, improving the bioavailability, drug efficacy, controlled release, targeting or protecting the active substance against severe conditions [1,2,3,4,5,6,7,8,9]. In contrast to various artificial nanomaterials, biopolymers possess several benefits as they exhibit biodegradability, biocompatibility and may also provide bioactive effects all by themselves [10]. These advantages render biopolymers highly interesting for implementing small molecules for drug delivering systems. The most widely studied bio-based polymers for such encapsulations, mainly for drug delivery in biopharmaceutical applications, are Polylactic acid (PLA), Poly-D,L-lactide-co-glycolide (PLGA) and Poly-ε-caprolactone (PCL), respectively [1,11].

In addition, the entrapment of substances in polysaccharides such as cellulose esters has been studied for various functions, again focusing on biomedical applications, as summarized in the review of Gericke et al. [12]. The biocompatibility of polysaccharides gives them a promising potential as nanocarrier material not only for drug delivery applications [13]. The incorporation of active compounds into polysaccharides nanoparticles such as cellulose ester nanoparticles can occur directly during nanoparticle formation through self-organization processes such as nanoprecipitation [12,14,15,16,17,18]. However, until today, in particular PLA [19,20], PLGA [20,21], PLGA-PEG [22] or PCL [23] have been studied for the entrapment of active substances via nanoprecipitation [24].

The nanoprecipitation process, also known as “solvent-displacement”, reshapes the polymer chains into nanoparticles by mixing a low concentrated polymer solution with a fully mixable non-solvent (e.g., water, if cellulose esters are being considered). Driven by mutual diffusion processes of the solvent and non-solvent, the polymer loses its solubility gradually and separates in the form of spherical particles [25]. Nanoparticles with different particle diameters are obtained easily by the nanoprecipitation as the polymer concentration is one parameter to tune the particle size, whereby larger particles are formed with increasing polymer concentration in general. However, the particle size is limited by the critical overlap concentration of the polymer in the solvent [12,18,25,26,27].

Compared to other methods of manufacturing polymer particles, such as emulsion polymerization, the nanoprecipitation allows a one-step nanoparticle preparation with simple equipment and without the need of emulsifying agents, toxic solvents or high-energy consumption. It requires pre-defined polymer molecules, which makes it even possible to gain biopolymer nanoparticles [12,20,28].

The formation of polymer nanoparticles during nanoprecipitation was first observed by Fessi et al. as early as 1989 [29]. However, the pioneering work for the preparation of nanoparticles from various cellulose esters, including cellulose acetate, was carried out by Hornig and Heinze in 2008 [25]. Later, Kulterer et al. [30,31] intensified research on the nanoprecipitation of polysaccharides, especially using cellulose acetate. Cellulose acetate is very suitable for nanoprecipitation without any additives, as these nanoparticles have a higher stability compared to other cellulose esters due to the higher glass transition temperature [32]. Cellulose acetate nanoparticles have also been used to entrap low molecular compounds, as outlined in the following.

In 2012, Kulterer et al. [31] investigated the entrapment of the hydrophobic fluorescent dye pyrene in cellulose acetate/carboxymethyl cellulose composite nanoparticles. Moreover, Schulze et al. [18] investigated the incorporation of three different hydrophobic dyes into cellulose acetate nanoparticles using dialysis nanoprecipitation in 2016. Cellulose acetate nanoparticles with antimicrobial activity were first examined by Liakos et al. [33,34] in 2016: They prepared cellulose acetate nanoparticles in combination with chemically bound lemongrass oil and also co-precipitated with copper ferrite in order to improve their antimicrobial activity against *E. coli* and *S. aureus*. In 2017 Mazumder et al. [32] investigated the preparation of nanoparticles of different cellulose acetate-based polymers by flash nanoprecipitation for the entrapment of hydrophobic antiviral drugs to enhance their solubility for pharmaceutical applications. The last decade has published a number of studies and demonstrators of cellulose acetate nanoparticles being loaded with active substances for a targeted release. However, taking a closer look at the literature also reveals a number of important open scientific questions that still need to be answered to further increase our understanding of such interesting bio-based nanoparticles as well as to eventually transfer these materials into appropriate candidates for distinct applications. For example, unanswered questions concern the entrapment of small molecules in cellulose acetate via nanoprecipitation and how these molecules influence the self-assembly of the polysaccharide chains during phase transfer from a “dissolved” state into a “solvent-free” state. What is the actual mechanism of encapsulation and how are the (entrapped) molecules distributed within or on the surface of the particles? Finally, can we tackle other possible uses than the often-targeted biomedical applications?

To answer these questions, in this contribution, we use two hydrophobic biocides, namely Triclosan and 4-Hexylresorcinol as model biocides, entrapping them into cellulose acetate nanoparticles by nanoprecipitation. The particle loading is characterized by nuclear magnetic resonance (NMR) studies and the particle morphology is studied by dynamic light scattering (DLS) measurements and Scanning electron microscopy (SEM) imaging, respectively, to deepen our understanding of the mechanism of entrapment. In order to evaluate the release of biocides from the nanoparticles, a standard zone of inhibition test is used. This test also shows the antimicrobial effectiveness of such nanoparticle-coated paper substrates, thereby targeting possible applications with antimicrobial paper materials. Although antimicrobial paper materials are of great general interest, the focus of the present work, however, focuses on the production of the particles and a fundamental understanding of the entrapment mechanism.

## 2. Materials and Methods

### 2.1. Materials

Cellulose acetate (CA) (Sigma-Aldrich, St. Louis, MO, USA, *M*_n_ 50,000 g/mol, DS 2.5), Triclosan (Alfa Aesar, Kandel, Germany, 99%), 4-Hexylresorcinol (Sigma-Aldrich, St. Louis, MO, USA, 98%), acetone (Brenntag, Essen, Germany, technical grade), Lumogen^®^ F Red 305 (BASF, Ludwigshafen, Germany), Inhibition plate (Axonlab, Reichenbach, Germany), *Bacillus subtilis*, pH 6.6), Rotilabo^®^ filter paper Type 601 (Carl ROTH, Karlsruhe, Germany) were used as model paper substrate, deionized water was used in all experiments.

### 2.2. Methods

#### 2.2.1. Nanoprecipitation

The fourfold volume of water is added dropwise to a stirred solution of 5 g/L cellulose acetate in acetone. For biocide-loaded nanoparticles, a biocide amount of 0, 0.10, 0.15, 0.20, 0.30, 0.40, 0.50, 0.60, 0.70 or 0.80 wt_Biocide_/wt_Polymer_ (*g*/*g*) is also added to the polymer solution prior to nanoprecipitation. For a size adjustment of the nanoparticles, experiments with a cellulose acetate concentration of 1.0, 1.5, 2.0, 2.5, 5.0, 6.0, 7.5 and 10.0 g/mL in acetone without addition of biocide are carried out. All nanoprecipitations were performed by adding the non-solvent water (approx. 15 mL/min) to a stirred (250 rpm) polymer solution. The resulting nanoparticles were transferred into an aqueous dispersion by centrifuging and re-dispersing (3 times, 15,000 rpm, 15 min). In order to obtain a defined concentration, the nanoparticles are freeze-dried and re-dispersed in defined volumes of water. The analogues synthesis of fluorescence dye-loaded nanoparticles was performed by adding 0.01 wt_Lumogen_/wt_Polymer_ (*g*/*g*) of Lumogen dye F Red 305 instead of biocide.

#### 2.2.2. Particle Characterization

^1^H-NMR spectra were recorded with 300 MHz Avance II NMR spectrometer from Bruker BioSpin GmbH. Therefore, the freeze-dried nanoparticles were re-dissolved in DMSO-d6 (Sigma-Aldrich).

Differential scanning calorimetry (DSC) is conducted with a DSC 3 from Mettler Toledo using STARe-Software for all evaluations. The measurements are performed using the TOPM Method with a heating rate of 0.1 K/min and pulse height of 0.0005 K.

Scanning electron microscopy (SEM) samples were prepared on silicon wafers and sputtered with 10 nm Pt/Pd (80/20) in argon plasma using a Sputter Coater 208 HR from Cressington. An SEM Type XL 30 from Philips, equipped with a field effect gun and a secondary electron detector was used with an acceleration voltage of 15 or 10 keV. For nanoparticle size determination, a minimum of 100 particles were measured using ImageJ evaluation software.

Dynamic light scattering (DLS) was performed on a Malvern Instruments Zetasizer Nano ZS90 at 25 °C. All evaluations were performed by the Zetasizer software. The particle size was approximated as the cumulants (z-average) size and the particle size distribution as the polydispersity index (PDI) according to ISO 22412:2017.

The critical overlap concentration (c*) of the cellulose acetate (*M*_n_ 50,000 g/mol, DS 2.5) is determined by viscometer measurements of a dilution series of CA in acetone (0–36 mg/mL) using a spindle viscometer from Brookfield.

#### 2.2.3. Paper Inhibition Assay

The paper inhibition assay is obtained by zone of inhibition tests performed with agar plates containing spores of *Bacillus subtilis* (Axonlab, Reichenbach, Germany, *Bacillus subtilis*, pH 6.6). A test paper with a diameter of 0.5 cm onto which defined amounts of biocide-loaded nanoparticles were applied by drop casting is placed on the agar surface. The plates are then incubated for 24 h at 37 °C and bacterial growth, or lack thereof, progress is documented by photographs. From these, inhibition zones were quantified using ImageJ [35].

## 3. Results and Discussion

### 3.1. Nanoprecipitation of Cellulose Acetate

**Figure 1:**
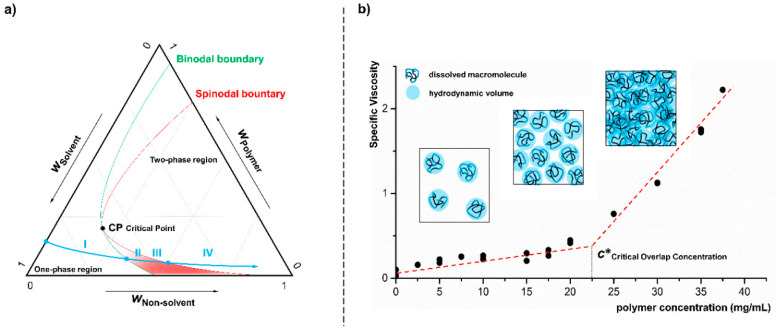
Schematic illustration of a ternary phase diagram (**a**) of polymer, solvent and non-solvent (mass fraction: *w*_i_) declares the addition of a non-solvent to a polymer solution. The blue arrow points to the transfer from a one phase region (I) into the two phase region (IV), thereby crossing both, the spinodal and binodal boundaries shown in green and red line-color, respectively. The graph (**b**) shows the determination of critical overlap concentration (c*) by viscosity measurements supplemented by a schematic depiction of the behavior of solvated polymer chains in the different concentration ranges.

We first prepared Cellulose acetate nanoparticles (NPs) via the solvent displacement approach, i.e., the so-called nanoprecipitation, whereby water as non-solvent was added dropwise to cellulose acetate polymer dissolved in acetone at concentrations well below the critical overlap concentration of the macromolecules. The concentration of the polymer was varied systematically to obtain a conclusive picture of the phase behavior. The addition of the non-solvent and its effect on the system can be illustrated in a theoretical ternary phase diagram (Figure 1a).

The continuous addition of the non-solvent water to a diluted polymer solution (cellulose acetate in acetone) leads to a degradation of the solvent quality and thus to a saturation of the system with cellulose acetate (I). With further addition of the non-solvent, the binodal boundary is exceeded and the system is converted to the metastable state. The three-component mixture of polymer, solvent and non-solvent is able to maintain the homogeneous form for a finite time despite formal supersaturation (II). Due to concentration fluctuations, however, nucleation and segregation of the system occurs at some point. At the latest, when crossing the spinodal boundary, the system demixes spontaneously into sol and gel (III). By further addition of non-solvent, the individual gel droplets consolidate and the final maturation of the particulate structures by condensation and coagulation takes place until the colloidal stability of the system is reached (IV). As a basic requirement for particle formation by nanoprecipitation a polymer concentration well below the critical overlap concentration (c*) is mandatory. As shown in Figure 1b in simplified form, up to this limit concentration, only independent solvated macromolecules are present in solution. At higher concentrations the hydrodynamic volumes of the polymers begin to overlap, i.e., the chains become entangled, which would lead to uncontrolled and statistic aggregation during precipitation. The critical overlap concentration of the cellulose acetate (*M*_n_ 50,000 g/mol, DS 2.5) dissolved in acetone, was determined by viscosity measurements to be approx. 22.5 mg/mL.

**Figure 2:**
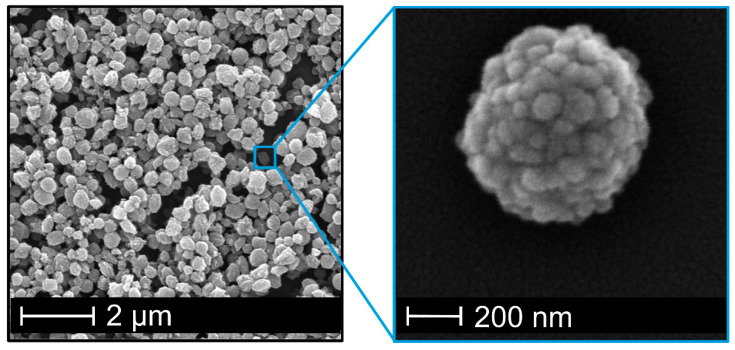
SEM image of cellulose acetate nanoparticles with strongly structured surface resulting of agglomeration of smaller substructures.

In the course of polymer nanoprecipitation, these two particle formation mechanisms are often referred to as “nucleation growth” and “nucleation aggregation” [11,12,18]. The nucleation growth mechanism (condensation) describes a continuous growth of initially formed nuclei by attachment of individual polymer chains from the surrounding medium. The nucleation-aggregation mechanism (coagulation), on the other hand, assumes an agglomeration of initially formed nuclei and very small particles. A closer look to the morphology of cellulose acetate particles (Figure 2), which we produced by nanoprecipitation here, suggests a dominant influence of the aggregation processes. The particles are characterized by a strongly structured surface and appear, at least externally, as the result of an agglomeration of smaller substructures.

**Figure 3:**
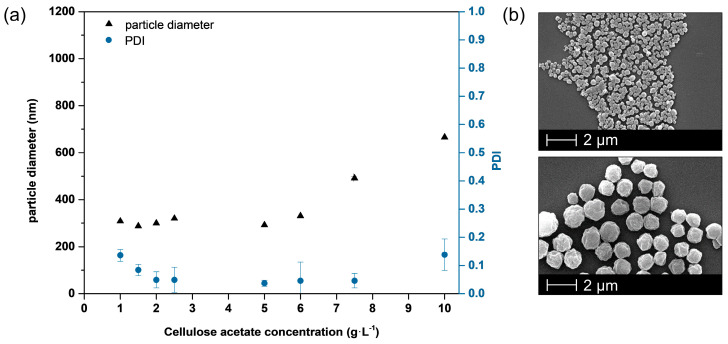
Adjustment of nanoparticle size by polymer concentration prior to nanoprecipitation process (**a**) leading to larger particles at higher concentrations without influencing the surface morphology (**b**).

For nanoprecipitations with polymer solutions below this c*, the polymer concentration affects the size of the resulting particles leading to increasing particle diameter at higher concentrations [18,25,26]. As can be seen from Figure 3a, the particle size of the so-formed cellulose acetate particles measured via dynamic light scattering increases with increasing polymer concentration. At the lowest polymer concentration of 1 g/L nanoparticles of approximately 300 nm are obtained whereby a concentration of 10 g/L is leading to a particle size of about 650 nm. The uniformity of particles is shown exemplary in SEM images of the smallest and largest particles (Figure 3b) and is expressed by polydispersity index (PDI) values (Figure 3a). The PDI varies from 0.04 to 0.14 and therefore show no fully monodisperse size distribution, which would imply a PDI value below 0.05 [36]. However, the size distribution is still small as the numerical value of the PDI theoretically ranges from 0.0 for perfectly uniform samples with respect to particle size to 1.0 for highly polydisperse samples with multiple sizes, whereas values bigger than 0.7 indicate such a broad distribution that the sample is not suitable to be analyzed by DLS technique [36]. Note, that the recommended PDI values depend on the application (e.g., food, cosmetics, pharmaceuticals), whereby values up to 0.3 are considered acceptable for drug delivery applications, for instance [36]. Therefore, we consider our PDI values of less than 0.15 to be very reasonable and consistent with values reported by others [12].

For the subsequent experiments on the entrapment of biocides in the nanoparticles, a polymer concentration of 5 g/L was chosen, resulting in small nanoparticles but with a more efficient process in terms of the quantities of solvent used. The latter thus corresponds to the highest concentration of nanoparticles that is produced after precipitation, which also reduces the effort required to process the dispersions.

### 3.2. Entrapment of Low Molecular Mass Hydrophobic Molecules into Cellulose Acetate Nanoparticles

Two hydrophobic biocides (Triclosan and 4-Hexylresorcinol) are selected as model compounds to investigate the entrapment and release mechanism. Both biocides exhibit broad-spectrum activity against a variety of microorganisms and were used in cosmetic products or in antimicrobial plastics, for instance [37,38,39,40,41]. Note that 4-Hexylresorcinol is also allowed according to present FDA regulations to be used in contact with food [41]. Both biocides have similar solubility properties to cellulose acetate, as they are basically insoluble in water and soluble in acetone (Table 1).

**Figure 4:**
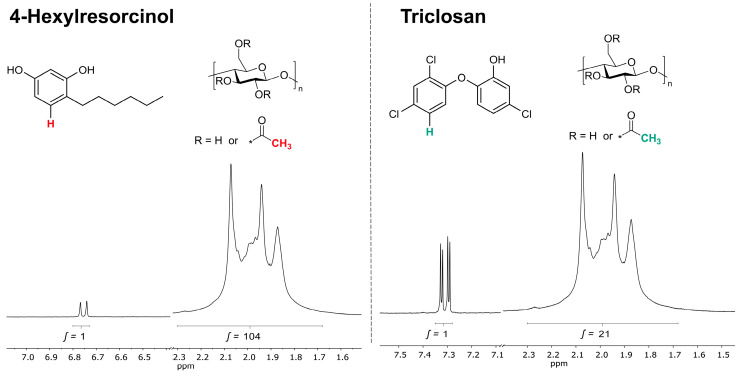
Signals of ^1^H-NMR spectra used for the calculation of the active substance content (**left**: 4-Hexylresorcinol, **right**: Triclosan).

The entrapment efficiency of both biocides is studied via ^1^H-NMR spectroscopy of re-dissolved (in deuterated solvent) freeze-dried nanoparticles, whereby the biocide content in the polymer solution prior to nanoprecipitation is varied. The biocide content is calculated from the ratio of a significant biocide peak to a significant cellulose acetate peak, taking into account the cellulose acetate molecular weight (50,000 g/mol) and degree of substitution (2.5). The peaks considered for this evaluation are shown in Figure 4. The complete ^1^H-NMR spectra of cellulose acetate, 4-Hexylresorcinol and Triclosan are shown in Supplementary Materials Figures S1–S3.

**Figure 5:**
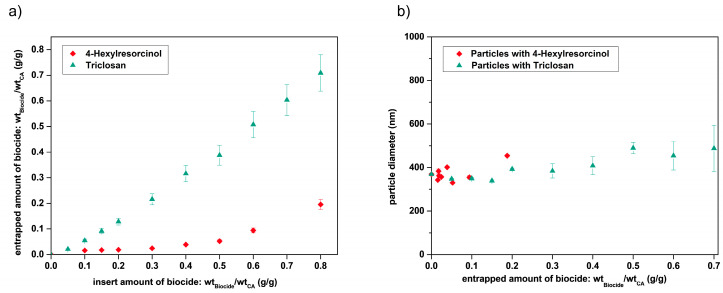
Amount of biocides (Triclosan, green or 4-Hexylresorcinol, red) entrapped into cellulose acetate nanoparticles (**a**) corresponding nanoparticle size determination of Triclosan (green) and 4-Hexylresorcinol (red) loaded cellulose acetate nanoparticles determined by DLS analysis (**b**).

The resulting particle loading for both biocides in cellulose acetate nanoparticles are shown in Figure 5a. The particles containing Triclosan or 4-Hexylresorcinol both show an increase in the entrapped amount of biocide with increasing biocide amount used throughout the nanoprecipitation process. This enables a precise adjustment of the biocide amount in the nanoparticles even though the efficiency varies strongly from biocide to biocide, as further discussed later. As shown in Figure 5b, neither of the biocides influence the particle size. Moreover, the morphology of these particles does not change with the entrapment of biocides (Figure S4), indicating that biocides do not influence the coagulation mechanism of the polymer chains.

### 3.3. Mechanism of Biocide Entrapment via Nanoprecipitation

The comparison of the two biocides Triclosan and 4-Hexylresorcinol demonstrates a strong dependence of the encapsulation efficiency on the molecular properties of the biocides and thus points to the question about the origin of these findings. We believe that Triclosan is entrapped more efficiently into the hydrophobic cellulose acetate nanoparticles via nanoprecipitation due to its much lower water solubility of 0.01 g/L compared to 4-Hexylresorcinol with a water solubility of 0.7 g/L and its higher hydrophobicity which can be expressed by the higher octanol-water partition coefficient (LogP Triclosan = 4.76; LogP 4-Hexylresorcinol = 3.34).

**Figure 6:**
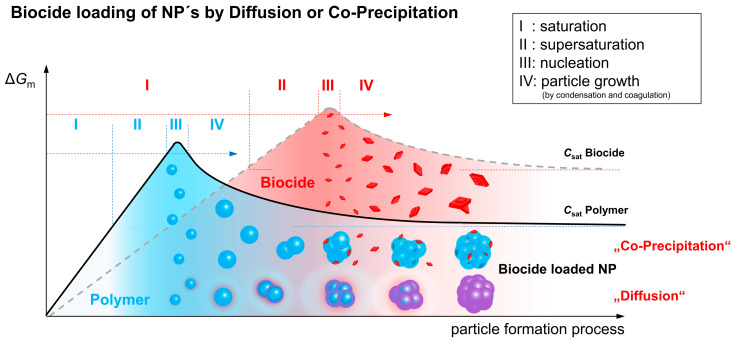
Schematic illustration of basic models of entrapment of hydrophobic compounds in hydrophobic polymers via nanoprecipitation.

In Figure 6, a schematic illustration is depicted, showing possible formation mechanisms of biocide-loaded particles during nanoprecipitation. When encapsulating hydrophobic compounds with hydrophobic polymers, two basic models can be distinguished, which are referred to as “co-precipitation” and “diffusion”, respectively.

As already described in the ternary phase diagram (Figure 1a), particle formation takes place via a phase separation process induced by the addition of a non-solvent to a dilute polymer solution. The added non-solvent leads to saturation (I) and supersaturation (II) of the polymer (blue) present in a sol phase before it finally starts to separate as a gel phase (III). The separating nuclei grow and/or aggregate (IV), and finally form the final polymer nanoparticles. In principle, this behavior, in particular stages I to IV, can also be described for the pure biocide (red). However, the curve is shifted in comparison to the polymer due to the individual solubility characteristics and the two-phase behavior is not associated with the separation of gel-formation but with crystal growth. If the polymer enters the phase separation stage earlier than the biocide, because of its significantly poorer solubility, the resulting polymer-rich gel droplets can serve as a solvent for the low-molecular weight component. Although the biocide has not yet reached the solubility limit in the remaining sol-phase, the molecules start to diffuse into the droplets due to their high affinity to the hydrophobic polymer [28] and adhering solvent. This diffusion process is supported by a continuous addition of the non-solvent and a thereby induced increasing biocide saturation in the sol. As a result of this “particle loading mechanism”, the biocide is molecularly dispersed within the polymer particle, however, a formation of a distribution gradient is also possible. In a reference experiment, a fluorescent dye instead of a biocide was used to prove that the hydrophobic low-molecular component is basically nanoparticle-bound after precipitation. In Figure S5 the corresponding confocal microscopic image of Lumogen F305 containing cellulose acetate nanoparticles is presented.

In the present investigations on the entrapment of Triclosan and 4-Hexylresorcinol in cellulose acetate particles, the latter formation mechanism “diffusion” is considered to be the most likely process taking place due to the following arguments. The addition of the non-solvent not only provides the driving force for the diffusion, but also offers sufficient time for the concentration equalization without driving the biocide into supersaturation (crystallization). The diffusion of Triclosan into the cellulose acetate nanoparticles is more pronounced in comparison to 4-Hexylresorcinol, which results in a higher particle loading (Figure 5). Due to the higher hydrophobicity and thus higher affinity to the hydrophobic cellulose acetate and the adhering solvent (acetone), expressed by a higher octanol-water partition coefficient (Table 1), an increased diffusion of Triclosan compared to 4-Hexylresorcinol into the forming particles confirms the established model of biocide loading by “diffusion”.

**Figure 7:**
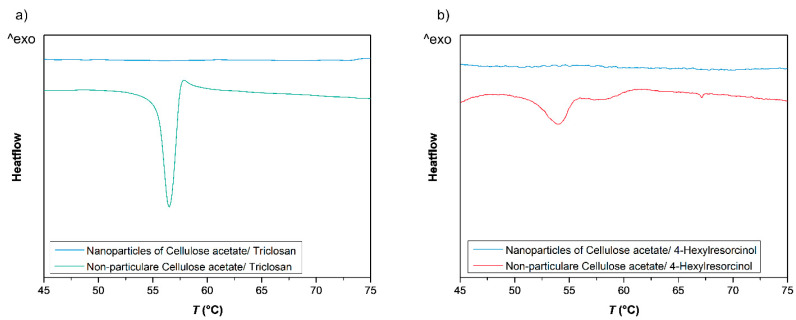
DSC measurement of cellulose acetate nanoparticles (blue) containing 0.2 g Triclosan/g cellulose acetate (**a**) or 4-Hexylresorcinol respectively (**b**) and non-particular cellulose acetate/Triclosan mixtures with identical Triclosan/cellulose acetate ratio (a, green) or 4-Hexylresorcinol/cellulose acetate mixtures (b, red).

Furthermore, as the data in Table 1 indicate, both biocides still have a relatively high solubility of 0.32 g/L for Triclosan and 1.93 g/L for 4-Hexylresorcinol in the final solvent composition (acetone: water, v:v, 1:4), hence, from a thermodynamic point of view, a biocide load according to the “co-precipitation model” would not be favored given these concentrations. Finally, DSC measurements (Figure 7) carried out with Triclosan (a) and 4-Hexylresorcinol (b) loaded nanoparticles compared to non-particulate cellulose acetate/Triclosan (a) or 4-Hexylresorcinol (b) mixtures confirm this model as well. The non-particular mixture shows a melting point at 57 °C of crystalline Triclosan or 53 °C of crystalline 4-Hexylresorcinol. In the cellulose nanoparticles, no crystalline biocide domains are revealed. These measurements strongly suggest that both biocides are dispersed amorphously in the nanoparticles and do not form significant amounts of crystalline domains, which would appear if all biocide is attached onto the particle surface, for instance. In addition, the latter again confirms the “diffusion” model, and opposes a separate precipitation, crystallization and adsorption onto polymeric nanoparticles of Triclosan.

### 3.4. Release of Biocide from Nanoparticles in Antimicrobial Zone of Inhibition Test

**Figure 8:**
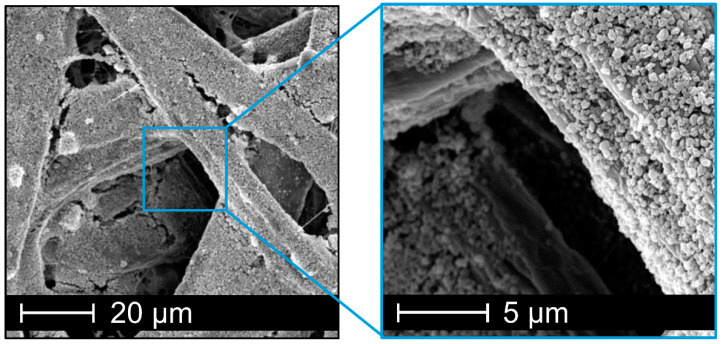
SEM images of model paper substrates coated with cellulose acetate nanoparticles with 3 g·m^−2^ for zone of inhibition test.

The biocide-loaded nanoparticles are coated onto model paper substrates to investigate their release and antimicrobial properties. This property, may become important, for example, in the design of paper-based packaging or construction materials where antimicrobial properties are of utmost importance to either ensure the material integrity of the paper (constructive) or to protect packed material such as food. The coating is achieved by a direct application of the aqueous nanoparticle dispersion (Figure S6) onto the paper substrates. The nanoparticles are attached to the fibers by this drop-casting method, as shown in SEM image (Figure 8). With an exemplary nanoparticle coating weight of 3 g/m^2^, the SEM images show a homogeneous coverage of the paper fibers (b); however, the pore cavities of the paper substrate are not filled with nanoparticles (a).

The release of the encapsulated biocides is tested by a standard zone of inhibition test, using the cellulase-producing microorganism *Bacillus subtilis*. This bacterium possesses a potential risk to paper materials due to its ability to digest cellulose. According to DIN EN ISO 20645 the zone of inhibition *Z* is calculated by the total diameter of sample and inhibition zone *D* and the sample diameter *d* (1):

(1) $$Z=\frac{D-d}{2}$$

**Figure 9:**
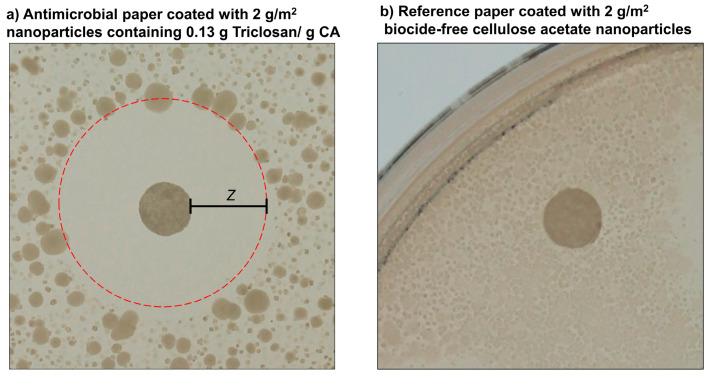
Paper substrates coated with 2 g/m^2^ Triclosan-loaded (**a**) or biocide-free (**b**) nanoparticles incubated on agar plates containing spores of *Bacillus subtilis* at 37 °C for 24 h. Picture (**a**) shows the release of Triclosan from nanoparticles (weight ratio: 0.13 g Triclosan/g CA) leading to an area expressed as zone of inhibition (*Z*) around the paper substrate due to diffusion of the biocide into the media, whereas biocide-free nanoparticles on a reference paper (**b**) show no suppression of bacterial growth.

The images in Figure 9 exemplarily show a test result of an effective antimicrobial paper (a), where no microorganisms grow on the paper substrate as well as in a specific area around the test paper due to a release of biocide into the media. In contrast, a paper coated with biocide-free nanoparticles does not cause an inhibition zone (b).

**Figure 10:**
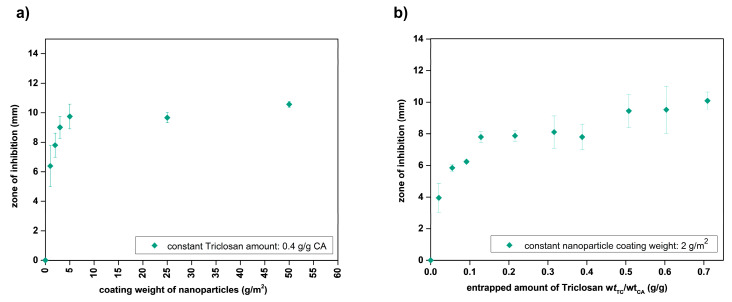
Zone of inhibition of Triclosan-loaded nanoparticles with variation of biocide loading concentration (**a**) and particle coating amount (**b**).

Since particle loading with Triclosan is far more efficient than with 4-Hexylresorinol, such particles were also investigated. As expected, the zones of inhibition saturate with increasing nanoparticles per paper disk (coating weight) at an otherwise constant biocide concentration (Figure 10a) as well as increasing amounts of entrapped Triclosan (Figure 10b).

**Figure 11:**
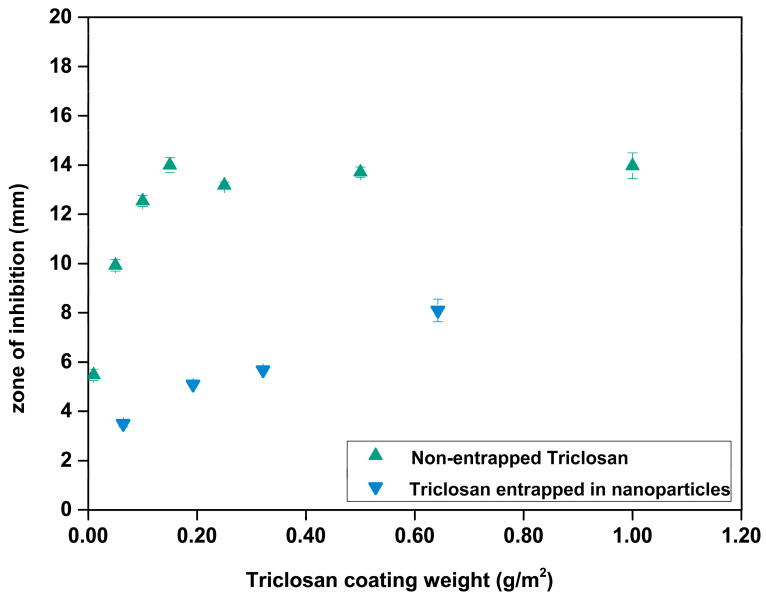
Comparing the release of Triclosan from nanoparticles (green) and pure Triclosan (blue) into the agar during the zone of inhibition test.

The direct comparison of identical coating amounts of Triclosan either entrapped into nanoparticles or applied purely (Figure 11) shows that Triclosan, apparently, is much slower released from nanoparticles than if supplied purely. The latter suggests that the nanoparticles hold back the Triclosan molecules and, as such, act as a barrier against the release of Triclosan.

This test demonstrates the attenuated release of Triclosan from cellulose acetate nanoparticles and thus enables the preparation of Triclosan particles with long-term biocidal activity. In addition to this increase in durability, such entrapment of biocides or other small molecules in polymeric nanoparticles also improves compatibility with the materials (e.g., paper), provides protection against destruction by environmental influences, such as UV radiation, and could even enable a controlled release.

Such controlled release could, for example, be triggered by particle-degrading microorganisms, which would induce a biocide release on demand. Cellulose acetate, along with other cellulose esters, is a very interesting nanoparticle material for the entrapment of biocides for antimicrobial paper properties, since paper degrading microorganisms (such as *Bacillus subtilis*) produce enzymes that do not only degrade the paper material cellulose, but could also degrade cellulose ester nanoparticles. The biodegradability of cellulose acetate can be adjusted to the respective application scenario by variation of the degree of substitution [42,43]. In the future, a long-term study of antimicrobial paper properties in real environmental conditions is planned in order to investigate the antimicrobial activity of the paper substrates in application-oriented conditions as a prospective construction material.

## 4. Conclusions

In conclusion, this contribution shows an efficient entrapment of biocides into nanoparticles using the bio-based polymer cellulose acetate. This nanoprecipitation process is well suited as it is a straightforward process with particle formation and biocide loading in a single step. The entrapped amount of biocide can be easily controlled by adjusting the amount of biocide used in the precipitation formulations, as is shown by means of ^1^H-NMR spectroscopy. The key requirement for the biocide entrapment is similar solubility behavior as for the polymer itself, meaning low water solubility and good solubility in an organic solvent (here: acetone) which is used as non-solvent/solvent system. Hence, the solubility parameters are of utmost importance, determining the nanoprecipitation mechanism. In addition, it has been observed that a decisive factor for a high packaging efficiency is a high hydrophobicity of the biocide, and thereby a high affinity to the hydrophobic polymer and the particle surrounding organic solvent. Biocides are entrapped into the nanoparticles by a “diffusion” mechanism during the precipitation process which leads to a homogenous distribution of the biocide in the particle without any crystalline enrichments of bioactive molecule. Finally, first conceptional studies reveal that paper substrates coated with aqueous nanoparticle dispersions exhibit antimicrobial properties, as shown by the model zone of inhibition tests. A possible field application of such particles could therefore include coatings, e.g., for antimicrobial papers in packaging applications, where a long-term effectiveness or even triggered release on demand of antimicrobial active substances is of great interest. Based on the understanding of the entrapment mechanism, using two model biocides, one may select and entrap other hydrophobic biocides, which can be specifically selected for a particular application, such as food packaging. Besides the here shown biocide 4-Hexylresorcinol, further interesting candidates are antimicrobial active natural compounds, such as the terpene carvacrol.

## Figures and Tables

**Table 1:** Substance data of biocides Triclosan and 4-Hexylresorcinol. Manufactory data ^(a)^ and determined via solubility measurements ^(b)^.

	Triclosan	4-Hexylresorcinol
Water solubility (g·L^−1^) ^(a)^	0.01	0.70
Octanol-water partition coefficient Log*P* ^(a)^	4.76	3.34
Solubility in final precipitation system (g·L^−1^) ^(b)^ (acetone: water, v:v, 1:4)	0.32	1.93

## Supplementary Materials

The following are available online at https://www.mdpi.com/2079-4991/10/12/2447/s1, Figure S1: ^1^H-NMR spectra of cellulose acetate, 300 MHz DMSO-d6., Figure S2: ^1^H-NMR spectra of 4-Hexylresorcinol, 300 MHz DMSO-d6., Figure S3: ^1^H-NMR spectra of Triclosan, 300 MHz DMSO-d6., Figure S4: SEM images of biocide-loaded nanoparticles with smallest (left) und highest (right) amounts of each biocide, i.e., Triclosan (top) and 4-Hexylresorcinol (bottom)., Figure S5: Confocal microscopic images of Lumogen dye entrapped in cellulose acetate nanoparticles, obtained via nanoprecipitation with a polymer concentration of 5g/L, Figure S6: Aqueous nanoparticle dispersion.
