# Supplementary material for: Entrapment of Hydrophobic Biocides into Cellulose Acetate Nanoparticles by Nanoprecipitation
Source: Nanomaterials (Basel). 2020 Dec 7;10(12):2447. doi: 10.3390/nano10122447 (PMC7762427; doi:10.3390/nano10122447)
Supplement: Supplementary file 1 [file nanomaterials-10-02447-s001.pdf]

nanomaterials

MDPI

Supplementary Materials

Entrapment of hydrophobic biocides into cellulose acetate nanoparticles by nanoprecipitation

Cynthia Cordt, Tobias Meckel, Andreas Geissler * and Markus Biesalski *

Macromolecular and Paper Chemistry, Technical University of Darmstadt, Alarich-Weiss-Straße 8, 64287 Darmstadt, Germany

* Correspondence: geissler@cellulose.tu-darmstadt.de; biesalski@tu-darmstadt.de

S1-S3: ^{1}H-NMR spectra

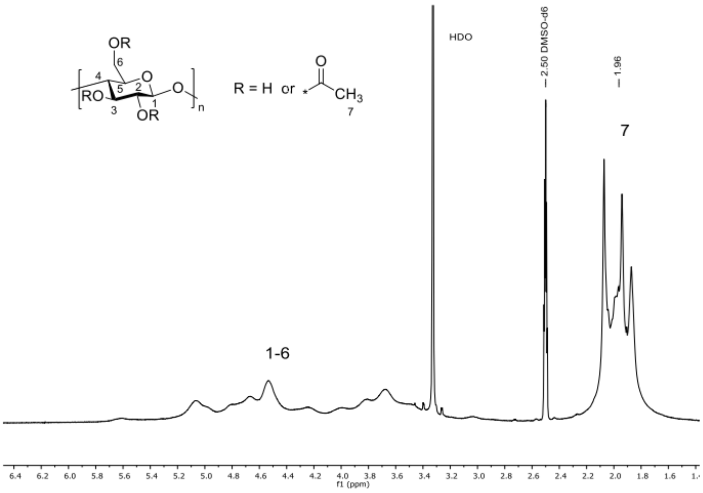

Figure S1: ^{1}H-NMR spectra of cellulose acetate, 300 MHz in DMSO-d6.

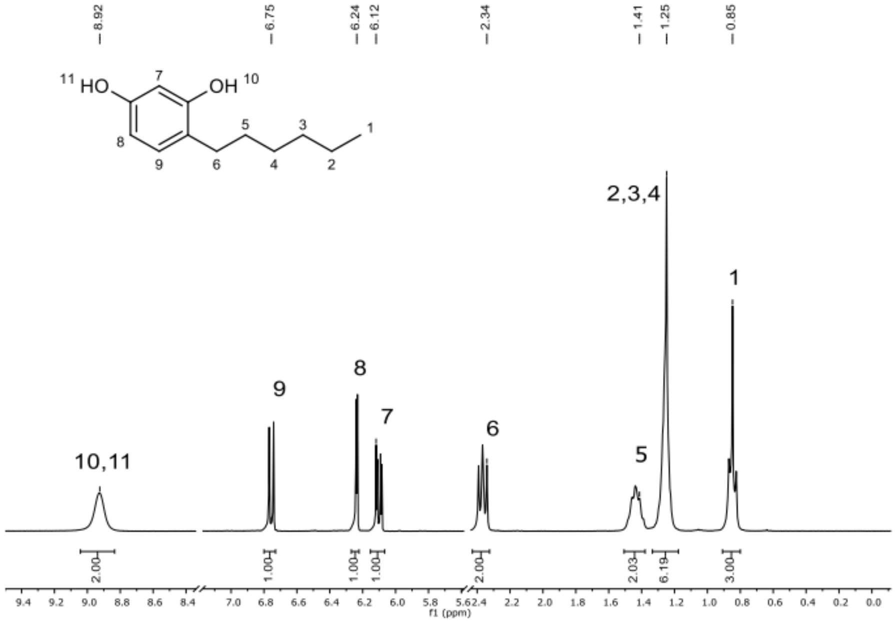

Figure S2: ^{1}H-NMR spectra of 4-Hexylresorcinol, 300 MHz in DMSO-d6.

nanomaterials

MDPI

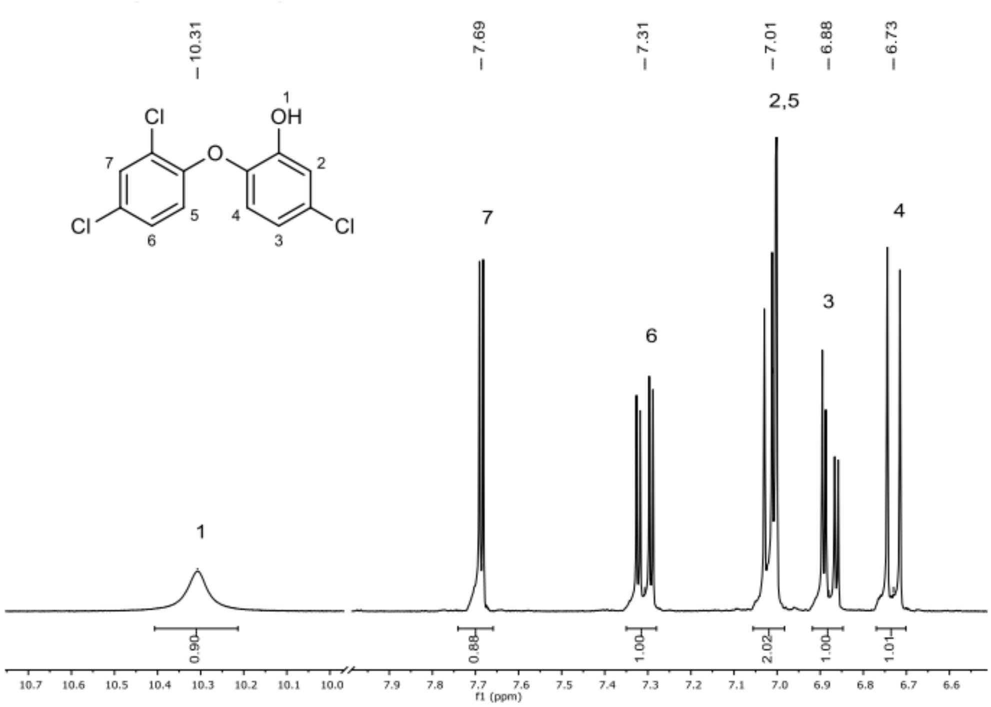

Figure S3: ^{1}H-NMR spectra of Triclosan, 300 MHz in DMSO-d6.

nanomaterials

MDPI

S4: Morphology of biocide loaded nanoparticles

The biocide content has no influence on the particle morphology, as the corresponding SEM images indicate. Figure S4 shows the particles loaded with Triclosan (upper row) and 4-Hexylresorcinol (lower row) with the smallest and highest amount of biocide respectively.

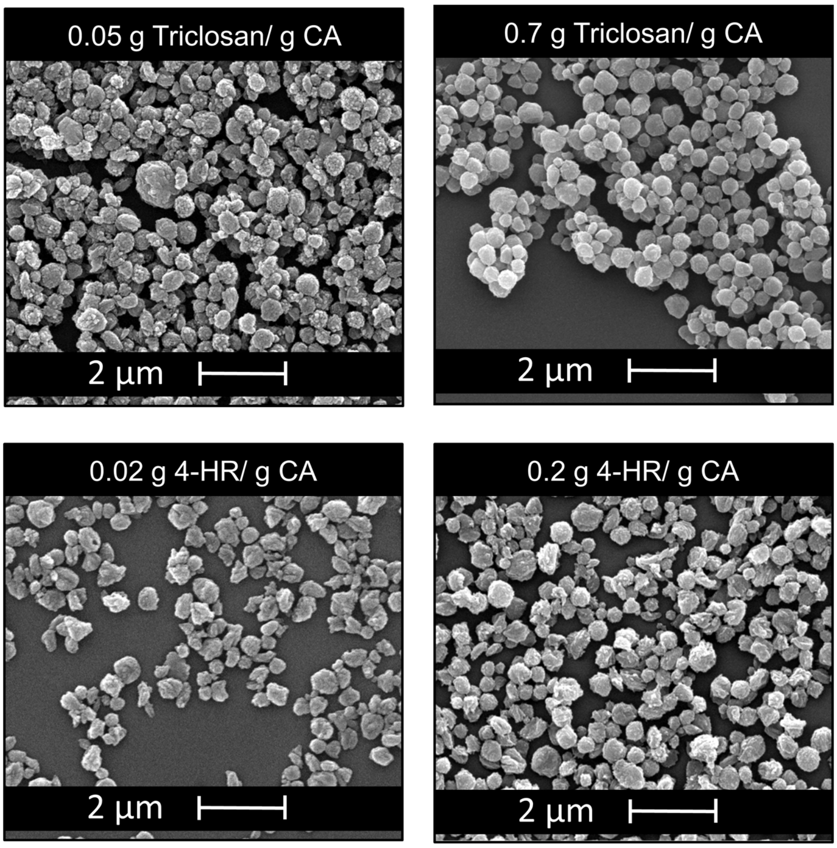

Figure S4: SEM images of biocide-loaded nanoparticles with smallest (left) und highest (right) amounts of each biocide, i.e. Triclosan (top) and 4-Hexylresorcinol (bottom).

S5: Entrapment of fluorescent dye into cellulose acetate nanoparticles

In addition to the biocide entrapment also a hydrophobic fluorescent dye (Lumogen F 305) was entrapped into cellulose acetate nanoparticles. A confocal image (obtained with a Leica TCS SP8 from Leica Microsystems) of the dye-loaded fluorescent nanoparticles, as shown in S5, gives proof of the dye entrapment into individual nanoparticles as the fluorescence of the Lumogen is clearly particulate.

nanomaterials

MDPI

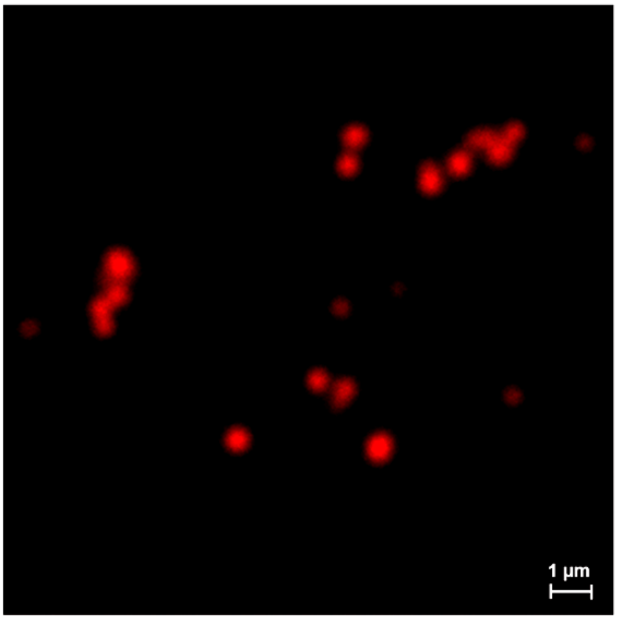

Figure S5: Confocal microscopic images of Lumogen F305 dye entrapped in cellulose acetate nanoparticles, obtained via nanoprecipitation with a polymer concentration of 5g/L.

It should be noted, however, that the diffraction-limited resolution of confocal microscopy, which is about 250 nm, does not allow an exact determination of the particle size and, even less so, the distribution of the dye within them. However, the fact that all particles have the same size apparent size well below 1 µm is sufficient to identify them as cellulose acetate nanoparticles loaded with Lumogen F305.

S6: Aqueous dispersion of biocide loaded nanoparticles

The nanoparticles can be coated onto paper materials directly out of an aqueous dispersion. Notably, although the nanoparticles sediment over time, no aggregation of the nanoparticles occurs, which makes them easily re-dispersible (Figure S6).

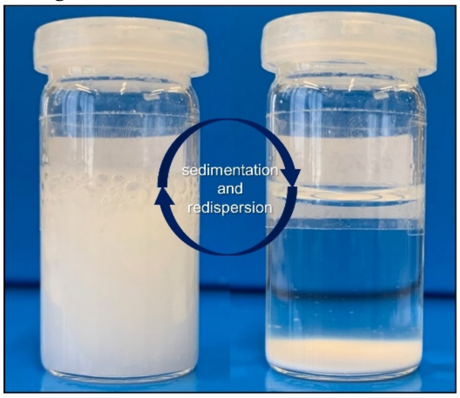

Figure S6: Sedimented biocide-loaded nanoparticles can easily be re-dispersed in aqueous medium by shaking before coating process.
